# The Impact of Resveratrol Supplementation on Inflammation Induced by Acute Exercise in Rats: Il6 Responses to Exercise

**DOI:** 10.22037/ijpr.2019.1100684

**Published:** 2019

**Authors:** Reza Vafaee, Hamidreza Hatamabadi, Hamid Soori, Mehdi Hedayati

**Affiliations:** a *Safety Promotion and Injury Prevention Research Center, Student Research Committee, Shahid Beheshti University of Medical Sciences, Tehran, Iran.*; b *Proteomics Research Center, Shahid Beheshti University of Medical Sciences, Tehran, Iran. *; c *Safety Promotion and Injury Prevention Research Center, Department of Emergency Medicine, Imam Hossein Hospital, Shahid Beheshti University of Medical Sciences, Tehran, Iran. *; d *Safety Promotion and Injury Prevention Research Center, School of Public Health, Shahid Beheshti University of Medical Sciences, Tehran, Iran. *; e *Cellular and Molecular Endocrine Research Center, Research Institute for Endocrine Sciences, Shahid Beheshti University of Medical Sciences, Tehran, Iran.*

**Keywords:** Training exercise, Inflammation factors, Trans-resveratrol supplementation, Rats

## Abstract

Severe physical activity leads to a sharp increase in free radicals, an oxidative stress, inflammation, and tissue damage. Resveratrol as one of the antioxidants can be effective in preventing the effects of oxidative stress. Therefore, the present study was aimed to evaluate the effect of trans-resveratrol supplementation and training exercise on inflammation-related factors. Sixty-four male Wistar rats were divided into six groups, each group consisting of 16 animals: 1) excursive + trans-resveratrol, 2) exercise group, 3) trans-resveratrol group, and 4) control group. Following the familiarization sessions, a more consistent protocol with an intensity of 65% vo2 max was performed for 12 weeks. Afterward, half of the mice in each group received acute exercise training with an intensity of 70-75% of vo2 max at the age of 20 weeks, until reaching the disability level. Finally, the levels of inflammatory markers were measured using special kits. Our findings depicted that inflammatory factors such as CPR, TNF-α, IL-6, and IL-7 were not affected by endurance protocol (*P *> 0.05), whereas, they were significantly increased by acute exercise training (*P *> 0.05). Additionally, we found that RES supplements led to a decrease in CPR and IL-6 levels, while not affecting TNF-α and IL-17 levels. According to available evidence, RES appears to have anti-inflammatory and protective effects during exercise by reducing inflammatory factors. Further studies are required to clarify the role of trans-resveratrol supplementation after exercise training.

## Introduction

Regular physical activity can have many health benefits, including reducing the risk of factors (cardiovascular disease, cancer and diabetes) that can lead to death. It has been clearly established that the contraction of skeletal muscle produces free radicals, where and intense and prolonged exercise can lead to oxidative stress on intracellular contents (1). The reactive oxygen species and nitrogen species play a key role in physiological processes such as signal transduction and training adaptation. It is noteworthy that muscular damage and fatigue arises when oxidative species are produced in abundance. Extreme exercise leads to oxygen species overproduction, resulting in oxidative stress (2). Fatigue is defined as mental or physical exhaustion, which is indicated to negatively affect the intensity of exercise, work performance, and social communication. Physical fatigue can be accompanied by a drop in physical performance. At least two mechanisms can be described for the occurrence of physical fatigue, which is referred to energy exhaustion and oxidative stress. 

Exhaustive or intensive exercises have resulted in excessive generation of reactive free radicals that can be associated with muscle tissue damage (3). Radicals are molecules or atoms that contain a single unpaired electron in their outer orbit. Due to their molecular instability, radicals are strongly reactive and can play a key role informing many harmful ooxidation reactions with proteins, lipids and cellular DNA, resulting in oxidative stress and cellular injury (4). Extreme exercise and muscle contraction have now been shown to increase the production of free radicals and other reactive oxygen species. In addition, current evidence suggests that a variety of reactive oxygen species can be the main cause of exercise-induced disturbance in muscle oxidation–reduction conditions in redox following exercise (4-6). 

Regarding the key role of various reactive oxygen species in developing oxidative stress and muscle fatigue, it should be taken into consideration that skeletal muscle fibers are involved in defensive mechanisms for decreasing risk of oxidative damage. Two main endogenous protective mechanisms including enzymatic and non-enzymatic antioxidants have been already defined to be implicated in reducing the detrimental effects of reactive species in muscle cells. On the other hand, it has been revealed that dietary antioxidants with endogenous antioxidants contribute to the formation of an antioxidant defense network (4). Based on current evidence, professional athletes involved in various sports activities exhibit a variety of physiological adaptations against chronic intense exercise conditioning. Both aerobic and anaerobic exercises are capable of producing free radicals that can cause acute oxidative stress. Oxidative stress can lead to oxidative damage and inflammation, which can be involved in more than 100 acute and chronic diseases. Attention has been paid to the adverse outcomes of these active oxygen species to the performance of sport and health of athletes after late 1970 (7).

The key role of polyphenols (*i.e.*, flavonoids) has been previously revealed in numerous biological activities, In particular, due to their antioxidant and antiradical characteristics, their structure*–*activity relationship has been widely investigated (8, 9). Also their structural needs for antioxidant and anti-inflammatory activity are thought to be different (8, 10). Trans-resveratrol (tRES) is recognized to be a phytochemical synthesized in various plant species such as grapes and peanuts. This active ingredient has been used in Japanese and Chinese traditional medicine in the form of powdery roots of the Polygonum cuspidatum plant to treat fungal infections, inflammation, blood pressure, allergies, and lipid disorders. TRES has been reported to be effective in protecting coronary heart disease and atherosclerosis by using moderate red grape juice (11, 12). In many studies, a variety of functional roles for resveratrol (RES) have been suggested, including antioxidants, platelet aggregation inhibitor, regulator of lipid, and lipoprotein metabolism and a vasodilator, but also play an inhibitory role in tumour development, indicating promise chemopreventive agent (12, 13). Research has identified the beneficial effects of trans-riversetrol on inhibiting oxidative stress and inflammatory actions from free radicals (14, 15). The question arises as to whether this supplement is effective on exercise adaptations, oxidative and inflammatory indicators in muscle injury during sports activities. The present study attempts to answer these questions. 

## Experimental

*Ethics Statements*

The study protocol was approved by the ethics committee under the leadership of the ministry of health and medical education, Iran*, *based on the guideline for the care and use of laboratory animals.

*Animals and design*

In this study, 64 male Wistar rats were purchased at the age of 6 weeks from Razi Research Institute of Karaj and were randomly divided into four groups (n = 16), an exercise + resveratrol group (n = 16), exercise group, resveratrol-treated group (n = 16), and a control group (n = 16). The animals were kept in a special animal lab on transparent polycarbonate shelves to reach the seventh week of the week. The animals have no limits on access to urban water and a standard diet until the start of the experiment to fix their metabolic status. The animals were first placed in 12-hour light conditions, followed by 12 h of complete darkness and relative humidity of 45% in a temperature-controlled room (22 °C ± 3 °C). Male Wistar rats housed 1 per cage and fed with commercial diet. The diets were manufactured by Pars Animal Feed Company. The exercise program was accordingly conducted during the most active time of the dark*/*light cycle (between 1:30 and 4:30 a.m.). Daily reports of dietary intake and weight of each rat were also recorded.

*The familiarization step*

At the seventh week, the familiarization step was conducted on a special rodent treadmill. Therefore, the rats were placed on a treadmill every 10 minat a speed of 5 to 10 m/min every two days. The last familiarization session was completed 16 to 18 h before the training program. The control groups were also placed on a treadmill 3 times a week that went off at a very slow speed for 10 to 15 min. Thus, the cage exit event take placed in the same way for all groups.

*TRES administration*

The rats were orally administered 10 mg/kg of tRES suspended in ethanol 2% during exercise for 21 continuous days. It is worth noting that applied dose was adjusted based on the rat weight to prove a constant dose. It should be noted that ethanol 2% in distilled water was given to the control group during the same period. 

*Endurance exercises*

The exercise protocol was increasingly started from the beginning of the eighth week, followed by exercising five days a week, at a speed of 10 m/min. Exercises continued to increase at speeds of 30 m/min for 60 min until the age of 19 weeks. Half of the mice were killed to determine the effect of the training after 48 h of the last exercise and 4 h of fasting. It should be noted that exercise intensity was estimated to be 65% Vo2max as described by Powers *et al.* (1993).

After this step, the remaining rats received an acute endurance exercise to assess the effect of the training on the maximal and minimal response at a speed of 25 m/min with a 10 ° slope.

After acute exercise, the rats were anesthetized by intraperitoneal injection of ketamine (30-50 mg/kg body weight) and xylosin (3-5 mg/kg). Using a heparin-treated 10*-*ml syringe, the blood samples were taken directly by cardiac puncture. The blood samples were then centrifuged at 3000×g for 15 min. Moreover, the plasma was rapidly removed from the cells, and immediately kept at −20 °C for further evaluations. Inflammatory factors were biochemically analyzed using commercial ELISA kits based on the protocols of the manufacturer. Inflammatory factors include CPR, TNF-α, IL-6, and IL-7.

*Statistical analysis*

SPSS software version 21 was used to analyze the data. To analyze the statistical differences among groups, one-way analysis of variance **(**ANOVA) was applied. Statistical significance was considered as *P* < 0.05. Tukey′s test was used to determine the place of difference.

## Results

*Change of Plasma C-reactive protein (CRP) Level*

*Effect of tRES and endurance training protocol* *(endurance protocol) on CRP plasma levels*

In order to evaluate whether tRES influenced inflammation in rates, we assessed the numbers of inflammatory markers. CRP is an important indicator of the inflammatory phase produced by the liver and found in blood plasma. This molecule has increased in response to inflammation and shows which tissues are inflamed due to injury.

**Figure 1:**
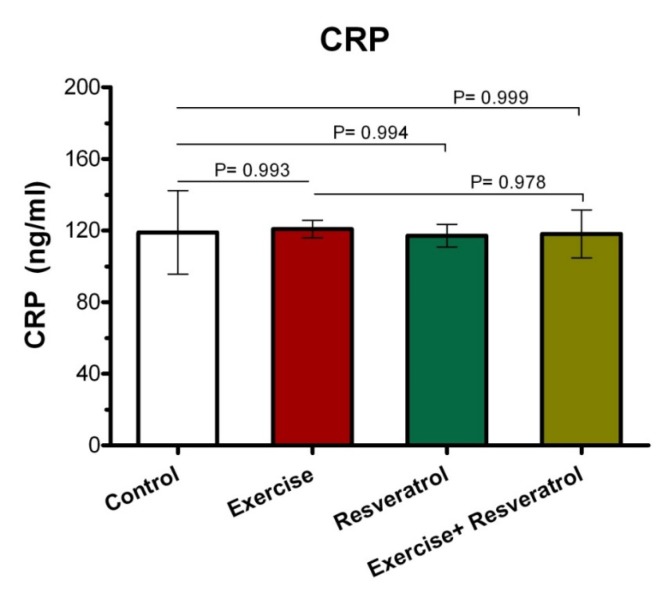
Effect of trans*- *resveratrol (tRES) supplementation and endurance exercise training on CRP plasma levels (in mg/L) in all groups.

As shown in Figure 1, after the implementation of the endurance exercise training, no significant difference was observed between the studied groups in terms of plasma CRP level.

*Effect of tRES and acute exercise training on CRP plasma levels*

**Figure 2:**
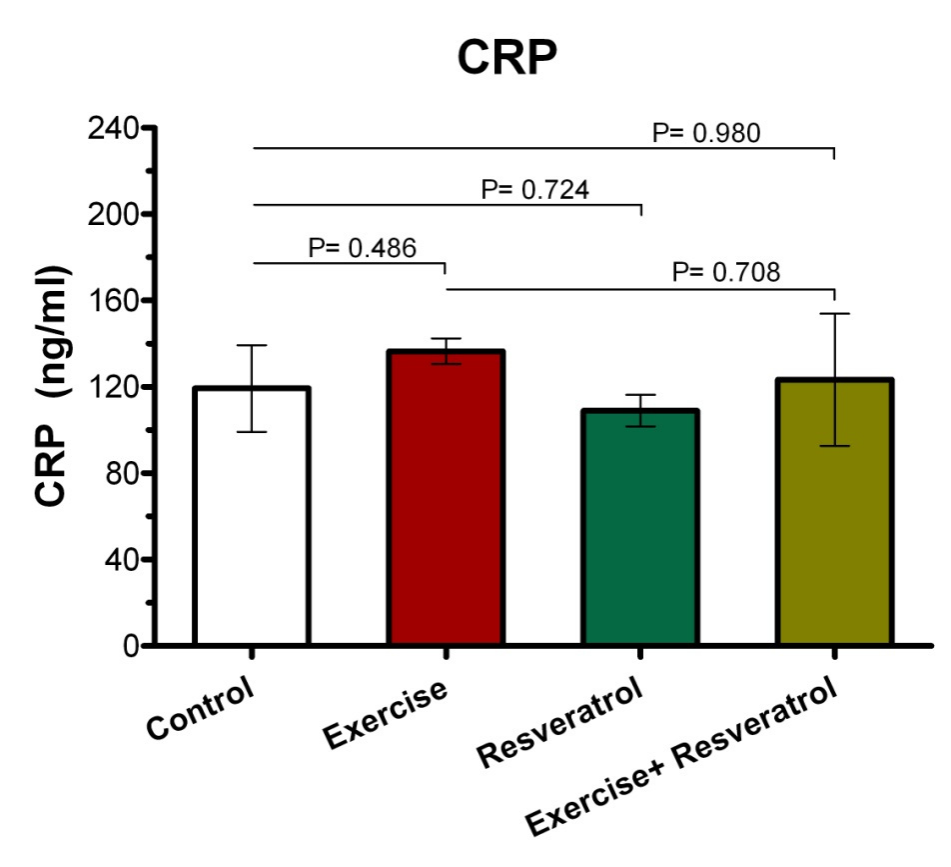
The ranges for CRP changes in response to acute exercise training (in mg/L) in all group.

CRP response is shown in Figure 2. Our findings indicated that there was no significant statistical difference between the groups in terms of plasma CRP level after acute exercise training.

*Comparing the level of CRP between the endurance exercise training and acute exercise training*

**Figure 3:**
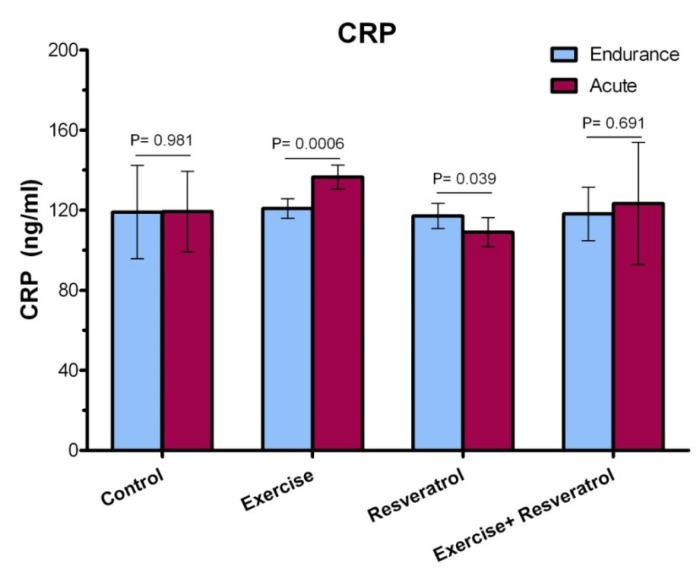
The ranges for CRP changes in response to endurance exercise training and acute exercise training (in mg/L) in all group.

Based on the data presented in Figure 3, a statistically significant increase in CRP level was observed after acute exercise training, when comparing with endurance exercise training group (*P* = 0.0006). There was no significant difference in the CRP between endurance exercise training group and acute exercise training group (*P* = 0.691).

**Figure 4:**
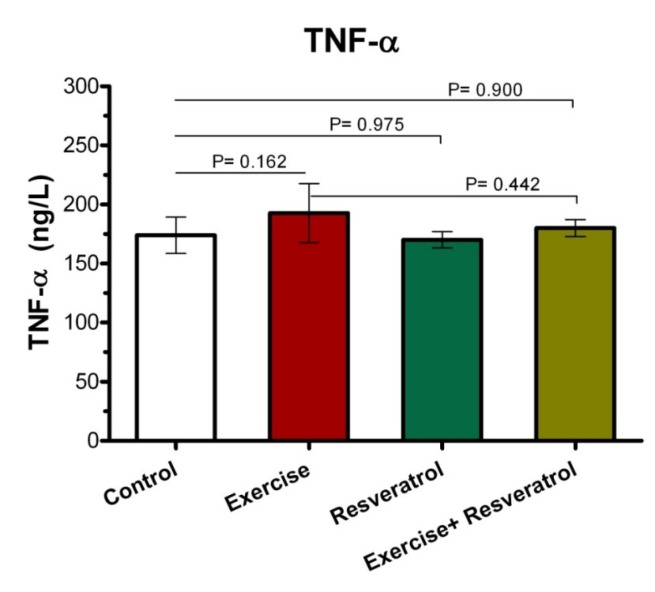
Comparison of TNF-α plasma level after implementing endurance exercise training protocol*.*

**Figure 5:**
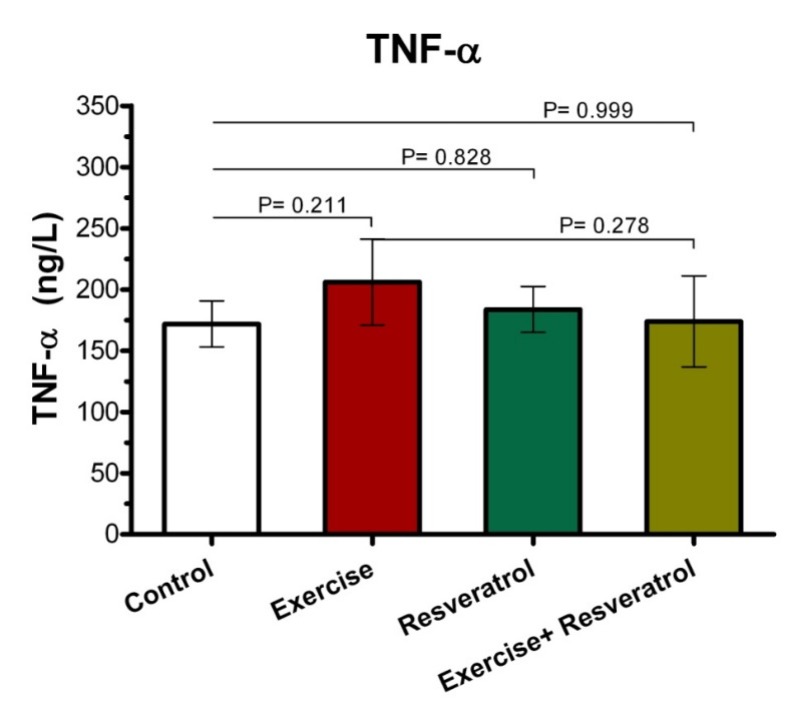
Comparison of TNF-α plasma levels after implementation of an acute protocol.

**Figure 6:**
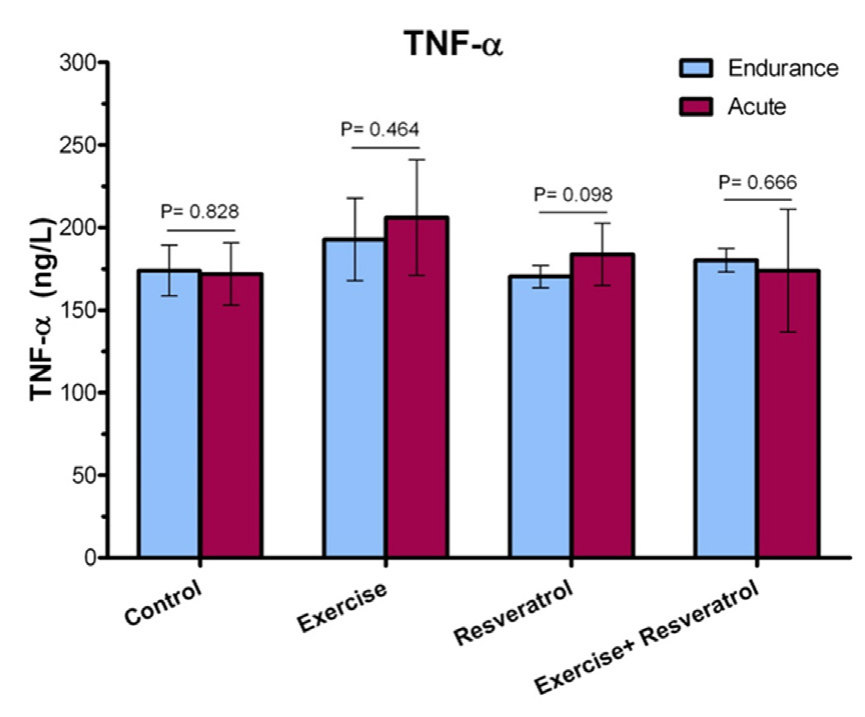
Comparison of TNF-α plasma levels after the implementation of the endurance exercise training and acute exercise training.

**Figure 7:**
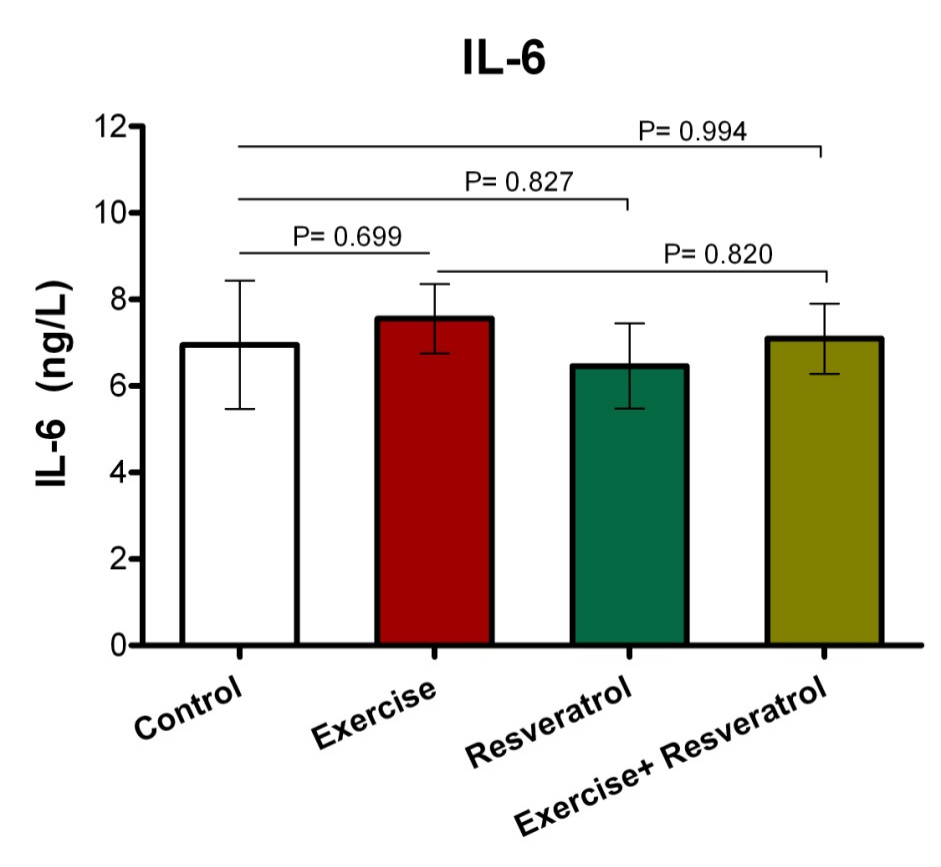
IL-6 response after the implementation of the endurance protocol.

**Figure 8:**
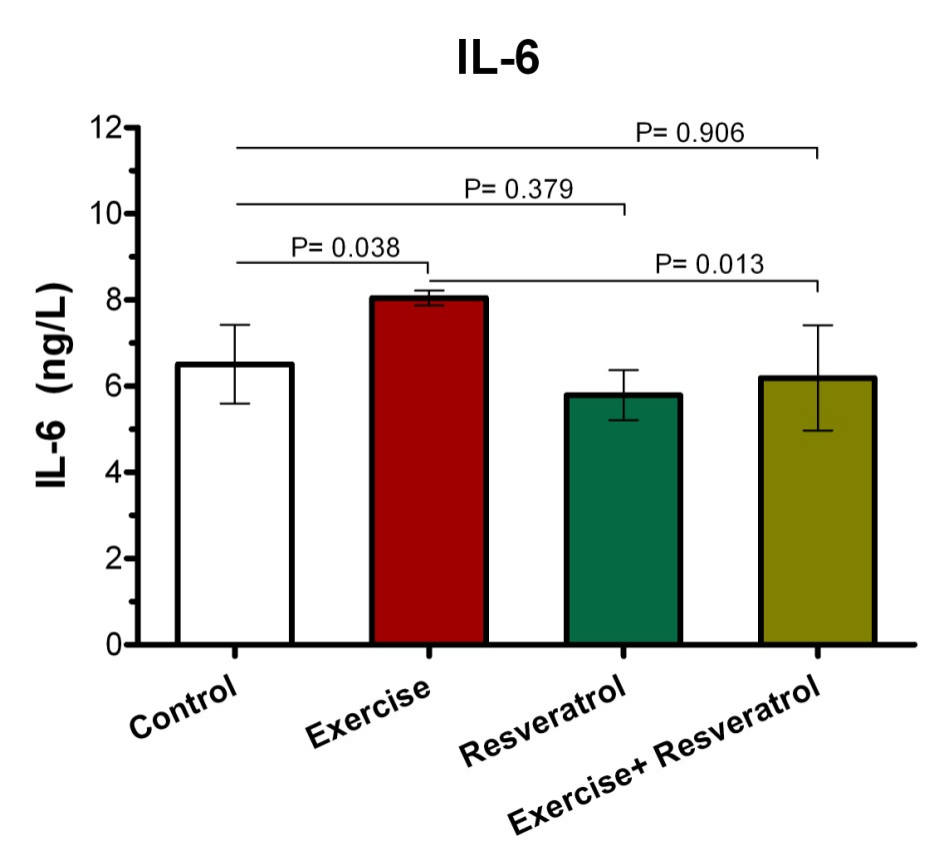
Comparison of IL-6 plasma levels among all groups after acute exercise training implementation.

**Figure 9:**
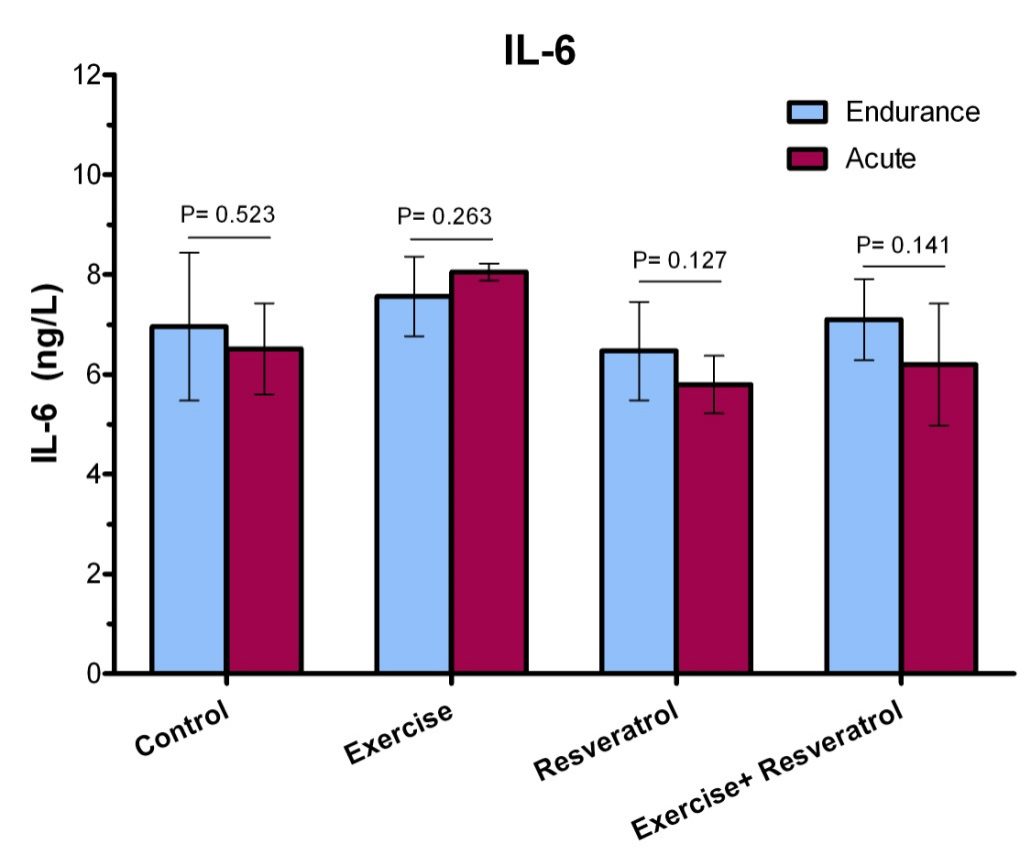
The ratio of IL-6 plasma level among studied groups after performing acute exercise training and endurance exercise training.

**Figure 10:**
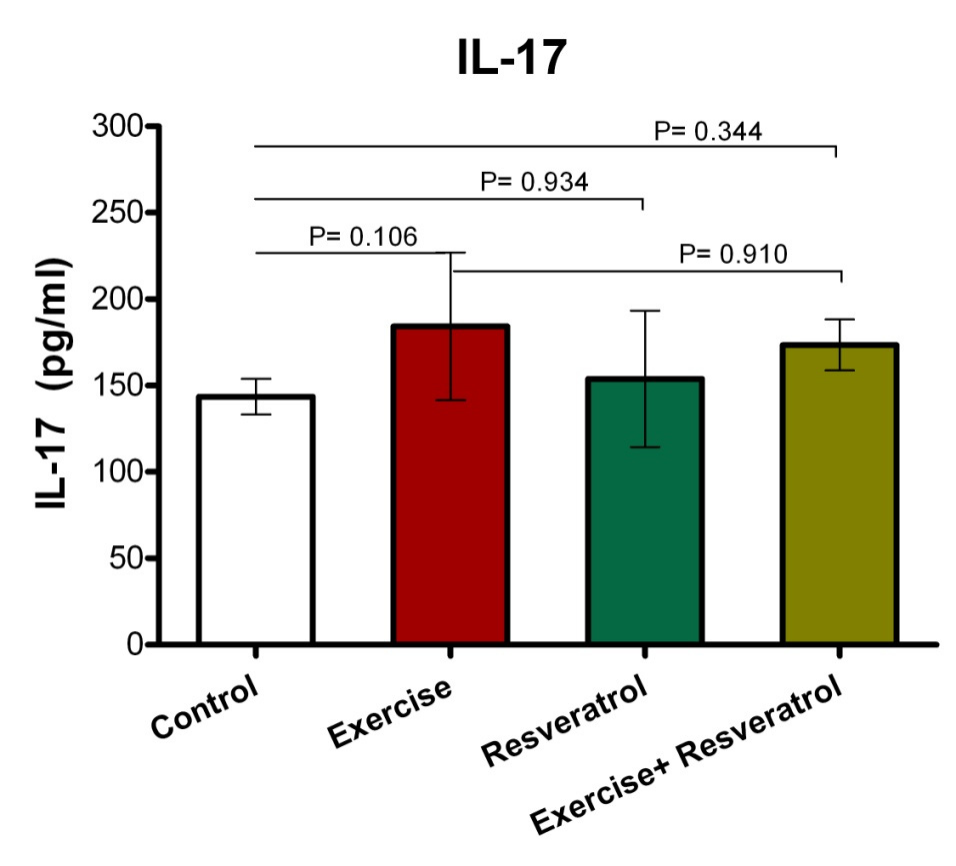
Comparison of IL-17 plasma levels after implementation of the endurance exercise training.

**Figure 11:**
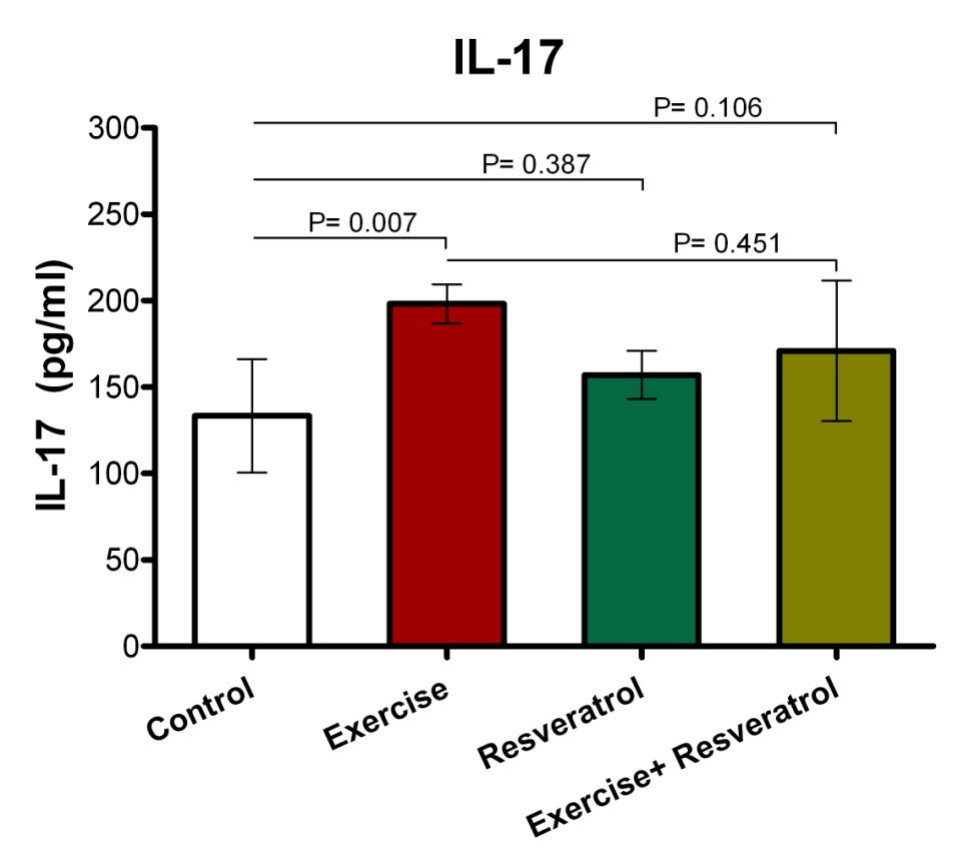
Comparison of IL-17 plasma levels after implementing acute exercise training.

**Figure 12:**
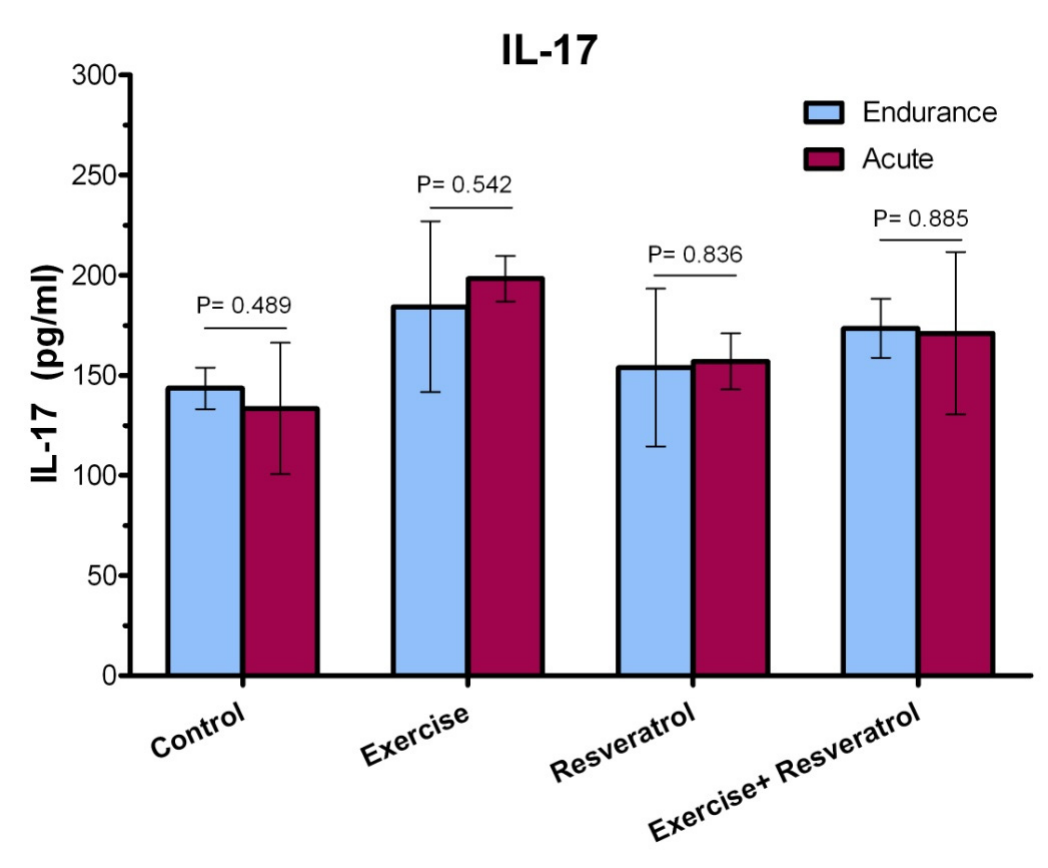
The ratio of IL-17 plasma level among different studied groups after performing acute exercise training and endurance exercise training.

*Change of Plasma TNF-α Level*

*The Effect of tRES supplementation and endurance exercise training on TNF-α Levels*

Tumor Necrosis Factor*-*α (TNF-α) is an inflammatory cytokine that is produced by NK cells and macrophages, which is one of the most important host defense mediators against viral and microbial infections. As presented (Figure 4), there was no significant difference in the TNF-α plasma level between all groups after implementing endurance exercise training protocol.

*Effect of tRES supplementation and acute exercise training on TNF-α*

Our results revealed that there was no significant difference in the TNF-α plasma level between the studied groups after implementing acute exercise training protocol (Figure 5). Interleukin 6 (IL-6) cytokines have both inflammatory and anti-inflammatory effects that produce tissue damage. In endurance athletes, the level of this cytokine increases when resting.

*Comparing TNF-α level between the endurance exercise training and acute exercise training*

Plasma TNF-α level (Figure 6) demonstrated no significant increase immediately after exercise between the endurance exercise training and acute exercise training.

*Change of Plasma Interleukin-6 Level*

*Effect of tRES supplementation and endurance* *exercise training* *on the Interleukin* *IL-6 plasma levels*

IL*-*6 have both inflammatory and anti-inflammatory effects, leading tissue damage. In endurance athletes, IL-6 response exhibited no significant difference in studied groups, where was not affected by endurance protocol (Figure 7).

*The Effect of tRES supplementation and acute exercise *training *on the IL-6 Level*

After performing the acute protocol, IL-6 plasma levels increased significantly in the exercise group compared to the control group (*P* = 0.038l; Figure 8). Interestingly, IL-6 plasma levels exhibited a significant decrease in the exercise + RES group as compared to the exercise group (*P* = 0.013), whereas there were no significant differences in IL-6 of rats in the in the exercise + tRES group when comparing with its level in the control group (*P* = 0.906; Figure 8).

*Comparison of IL-6 levels between acute exercise training and endurance exercise training*

As presented in Figure 9, IL-6 response exhibited no significant change between acute exercise training and endurance exercise training in the studied groups.

*Change of Plasma Interleukin-17 Level*

*Effect of tRES supplementation and endurance exercise training on the Interleukin IL-17 plasma levels*

IL-17 has been described as an inflammatory cytokine that represents inflammation of the musculoskeletal system. After the implementation of endurance exercise training, no significant difference in IL-17 plasma levels among groups was depicted in the current study (Figure 10).

*Effect of tRES supplementation and *acute *exercise *training *on the surface of IL-17*

Based on the data depicted in Figure 11, the level of plasma IL-17 in the exercise group showed a statistically significant increase as compared to the control group after performing the acute protocol (*P* = 0.007). Furthermore, there were no significant differences in plasma IL-17 level in the exercise + tRES group compared to the control group (*P* = 0.106).

*Comparison of IL-17 level in plasma between exercise training and endurance exercise training*

Based on the data presented in Figure 12, IL-17 response exhibited no significant change between acute exercise training and endurance exercise training in any groups, where was not affected by intervention.

## Discussion

Physical activity, depending on its type, duration, and severity, has several effects on the human immune system. Previous investigations have revealed that cytokine production is altered by a range of physiological stimuli including intense exercise, stress hormones, energy crisis, and oxidative stress (17, 18). For instance, regular mild exercises stimulate the body′s resistance to infections such as upper respiratory tract infection by enhancing the level of activity of the immune system, while extreme sports significantly reduce the resistance of the body to such infections (19).

Evidence suggests that intense exercise can increase the pre-inflammatory cytokines. The presence of several cytokines in urine and blood after exercise indicates that expression of cytokines is possible in response to exercise. Very heavy sport also lead to an increase in the number of stress-induced inflammatory cells, such as lymphocytes, monocytes and neutrophils, all of which can release a wide range of cytokines and growth factors (20-22). Evidence suggests that the production of several pro-inflammatory and anti-inflammatory cytokines increases rapidly in response to intense physical activity and other types of stress. Various studies emphasize that a variety of factors such as athlete′s readiness, hydration status and dehydration, as well as the duration and intensity of exercise, contribute to the increase in the levels of pre-inflammatory and anti-inflammatory cytokines.

On the other hand, the anti-inflammatory effects of tRES have been depicted in several studies (23-28). Recently, a study has shown that the use of RES could contribute to anti-inflammatory effects by increasing the level of, in the present study, there was no significant (27). Accordingly, the possible effects of RES on several inflammatory factors such as CPR, TNF-α, IL-6, and IL-7 were investigated in the current study.

PR molecule is one of the acute*-*phase proteins (APPs) that are secreted by T cells and macrophages following IL-6 secretion and its levels will rise in response to inflammation and infections. In this study, there was no significant difference in CPR plasma levels between exercise and control groups. These findings are consistent with the findings of some previous studies. For example, a study by Risoy *et al.* (2003) indicated that CPR protein has exhibited no significant change in athletes compared to normal population after performing heavy endurance exercise (29). Niess *et al.* reported that plasma CPR level demonstrated only minor change in male subjects after intensive endurance exercise on the treadmill (30). Recently, Tunc-Ata *et al.* (2017) have shown that acute and chronic exercise in Wistar Albino male rats did not change the CPR level, where responses to muscle damage of IL-6 and CRP have not been found to be affected by both mentioned activities (31).

However, in the present study, a significant increase in CPR level was observed in the acute training group compared to the endurance training group. Indeed, the intensity of exercise seems to be an important factor in CPR changes induced by exercise. On the other hand, some previous studies have determined that resistance and endurance training could reduce the level of CPR in healthy subjects and/or patients that are involved with CPR. For example, Soheili *et al.* (2009) have shown that the endurance exercises program, including running at a gym with 60-70% of maximum heart rate for 8 weeks and 3 times a week, significantly reduced CPR in older men (32). Another study has revealed that resistance exercise program in women with multiple sclerosis has reduced CPR levels (33). It should be noted that important factors such as genetic history, overweight, tobacco use, alcohol, and low exercise could affect CPR levels in different individuals. The linking mechanisms of obesity-induced inflammation are oxidative increases and CPR increase in obese individuals. Therefore, exercise training may reduce CPR levels appropriately by decreasing fat mass through direct effects on the inflammation process (34). Previous studies have shown that anti-oxidant RES results in lower CPR levels (35). However, in the present study, there was no significant change in the level of CPR in the tRES group. However, the increased level of CPR in the acute group + tRES was again reached to the level in the endurance exercise group + tRES. Based on these observations, with the increase in the intensity of exercise, the level of CPR was more affected by the use of tRES.Moreover, the findings of the current study suggested the effect of tRES on CPR plasma level change. McAnulty *et al.* (2013) depicated that CRP plasma level elevated markedly after exercise, where were not affected by RES and quercetin supplementation. A study demonstrated that RES supplementation (40 mg daily for 6 weeks) can be associated with a remarkable decrease of CRP in healthy subjects as compared to placebo (36). Ghanim *et al.* (2010) also revealed suppressive effect of RES on oxidative and inflammatory stress.TNF-α is a cytokine with multiple functions that play a key role in systemic inflammation and is involved in acute phase reaction (37). In the current study, plasma TNF-α level in exercise and control groups did not show any significant changes. These observations coincide with the findings of some of the previous studies. For instance, it has been demonstrated that TNF-alpha exhibited non-significant reductions after resistance training in individuals with multiple sclerosis (33). No training-dependent changes were observed in male Wistar rats for TNF-α based on the use of a ladder-climbing protocol after resistance exercise training (38).

 On the other hand, some observations have shown that exercise and physical activity can increase TNF-α levels (39, 40). A study by Zaldivar *et al.* emphasized that the exercise program, including a 30-min session of heavy cycling exercise, greatly increased TNF-α expression (41). Regarding the effect of tRES on TNF-α levels, our finding revealed that there was no significant change in plasma TNF-α level in the tRES group. 

On the contrary, most studies have depicted that the use of RES can moderate or decrease TNF-α levels, especially after exercise (42-45). For instance, it has been revealed that the use of RES in Wistar rats with elevated levels of TNF-α resulted in a significant reduction in TNF-α levels (42). Moreover, Tung *et al.* suggested that the RES supplementation in old mice liver significantly reduced TNF-α level (45). It has been reported that a supplement containing 20% RES for 6 weeks has led to a significant reduction in TNF-α levels in male professional basketball players (44). Additionally, another investigation also emphasized that the RES supplementation reduced and regulated the increased level of TNF-α after a physical fitness test on military firefighters (43). Recently, Hajighasem *et al.* also found that the RES supplementation alone or in combination with continuous exercise significantly reduced levels of TNF-α and malondialdehyde in rats suffering from non-alcoholic fatty liver disease (46). However, in consistent with our findings, Jeong *et al.* found that a moderate exercise training was much more effective than RES supplementation for reducing TNF-α levels (Jeong *et al.*, 2015). Overall, the effects of exercise and RES supplementation on TNF-α change may be related to several factors, such as type and duration of exercise, and the type of model examined, as well as timing, and dosage of RES supplementation, etc. 

IL-6 exert both inflammatory and anti-inflammatory properties. The main sources of IL-6 production are active macrophages, fibroblasts, and endothelial cells, and its main actions are described on cells, including the growth of T and B cells, stimulation of the acute phase response. Furthermore, IL-6 expression level within the muscle could be increased, when intramuscular glycogen reaches its critical status (34). In the present study, changes in the level of IL-6 were not found by performing the endurance exercise. These findings are consistent with the results of many previous studies which have shown the relationship of exercise training with increased IL-6 levels (47-49). Ahmadi *et al.* reported a significant increase in serum IL-6 levels after exercising OLEMPICS (49). In addition, Zaldivar *et al*. (2006) observed that IL-6 levels have increased significantly after a 30-min cycling exercise (48). However, in some studies, changes in IL-6 levels have not been found after exercise training (33, 50 and51). Based on the results of a study, moderate-intensity resistance excursive training with 3 days/week for 8 weeks could not change CK, TAC, IL-6, and IL-1β levels (51).

In addition, the study by Risoy *et al.* (2003) demonstrated that there was a very slight change in the level of IL-6 after performing heavy endurance exercise (50). Based on observations obtained from studies, the increase in IL-6 during physical activity and exercise is associated with various factors such as duration and intensity of exercises, involved muscle mass, and individual endurance capacity. In fact, more than 50% of changes in serum IL-6 after exercise can be explained alone by the duration of the exercise (52). It can be described that exercise can increase up to several times after endurance exercise at plasma and urine levels of IL-6 (53, 61). Additionally, age can also be considered as an important factor in determining the response rate of IL-6 to physical activity.

The effects of RES supplementation on the IL-6 level have been shown by various studies (54). For example, Shahidi *et al.* (2017) depicted that various concentrations of RES was led to decreased IL-6 secretion by human fibroblast cells. The findings of our study revealed that IL-6 in the acute exercise group + tRES decreased significantly compared with the acute exercise *-*alone group and reached the level of the control group. These findings are consistent with some previous studies (43, 44 and 54) and are in contrast with others (45).

The study by Zahedi *et al.* showed that supplementation containing 20% tRES after 6 weeks significantly reduced plasma level of IL-6 in male professional basketball players (44). Macedo *et al.* (2015) also found that RES supplementation play its anti-inflammatory effect throgh reduction of IL-6 and TNF-α levels after a physical fitness test among Brazilian military firefighters (43).

In contrast, it has been previously demonstrated in animal experiments that the use of RES in old mice (without exercise activity) did not significantly change the level of IL-6 (45). Overall, RES is likely to reduce or moderate the increased levels of IL-6, where are associated with physical activity and may not have much effect on IL-6 changes that are related to other factors such as age. However, it is better to assert this hypothesis by further studies in the future.

The cytokine IL-17 is a pro-inflammatory cytokine that plays a key role in mobilizing granulocytes such as neutrophils and promoting inflammation, and also regulates the function of T cells. Another subgroup of T cells called Th1, has been identified that can produce IL-17, IL-6, and TNF-α cytokines. In the present study, although there was no change in IL-17 level with endurance exercise protocol; however, after acute exercise activity, there was a significant increase in IL-17 levels compared to the control group. These events are consistent with the findings of the previous studies. 

Duzova *et al.* showed that IL-17 levels in strenuous exercise group (Eight week old rats) were significantly increased compared to the control group. They also observed a strong negative association between the levels of IL-17 and IL-6 (56).

On the other hand, it has been suggested that endurance exercise for one hour with a 60% VO2max intensity on the treadmill did not significantly change the levels of IL-17 and CPR (55). It seems that the increase in IL-17 is correlated with the intensity of the activity during exercise and can be one of the causes of inflammation in the airways caused by exercise (56). Although the effects of RIS on the IL-17 level have been studied in several studies, to best of our knowledge, the researcher has not yet studied the effects of RIS on changes in IL-17 levels after exercise training (57). Based on the data present in our study, unlike IL-6, IL-17 levels in the exercise group + tRES were not significantly different in comparison with the exercise group. This finding is consistent with previous evidence. For example, Tung *et al.* (2015) have depicted that there was no significant change in the level of IL-17 in the old mice (without exercise activity) following the RES supplemntion (45). In different studies the association of IL 6 and CRP is mentioned. Ferrari *et al.* in a three- year follow up explains the effect of IL 6 on CRP as a promoter (58, 61). A review study by Esenva and *et al.* demonstrated that chronic inflammation leads to increase of CRP and IL-6 (59). In this study acute exercise cause significant elevation in IL 6 level and also acute exercise elevated mean CRP without achieving significant level. This finding in accordance with other studies which showed “other inflammatory factors can stimulate CRP” indicates to the weakened relationship between interleukin-6 and CRP (60).

## Conclusion

The results of our study revealed that the endurance protocol had no effect on levels of inflammatory factors such as CRP, TNF-α, IL-6 and IL-7, while the acute treatment protocol significantly increased the levels of CRP, IL-6 and IL -l7. RES supplementation resulted in moderating or decreasing levels of IL-6, but did not affect TNF-α and IL-17 levels. Based on data present here, RES seems to have anti-inflammatory and protective effects during exercise by decreasing levels of IL6.
